# Do women with statin-related myalgias have low vitamin D levels?

**DOI:** 10.1186/s13104-015-1356-9

**Published:** 2015-09-17

**Authors:** Margo Minissian, Megha Agarwal, Chrisandra Shufelt, Puja K. Mehta, Talya Waldman, Greg Lentz, Galen Cook-Wiens, Jo-Ann Eastwood, C. Noel Bairey Merz

**Affiliations:** The Barbra Streisand Women’s Heart Center, Cedars-Sinai Heart Institute, 127 San Vicente Blvd AHSP Suite A9306, Los Angeles, CA 90048 USA

**Keywords:** Vitamin D, Statin, Myalgia, Statin intolerance, Alternative statin dosing

## Abstract

**Background:**

Statin intolerance is often due to myalgias. Severe vitamin D deficiency is characterized by musculoskeletal pain. We hypothesized that statin-intolerance is associated with vitamin D deficiency.

**Objectives:**

To determine whether there is an association between statin-intolerance and vitamin D deficiency in a retrospective observational analysis.

**Methods:**

We evaluated 20 female patients with prior myalgia-related daily dose statin intolerance on an alternative day statin dosing protocol of twice weekly for 4 weeks followed by advancement to daily dosing, as tolerated. Fasting baseline and follow-up lipid and 25-hydroxy-vitamin D (25-OHD) levels were obtained by chart review.

**Results:**

The group median age was 61 ± 13 years old and BMI was 27 ± 7 kg/m^2^. Women who remained on alternative day statin dosing (n = 16) compared to women on daily dosing (n = 4) had a significantly lower group mean 25-OHD (mean 29 ± 11.23 vs. 47.5 ± 23.53 ng/ml p = 0.0307 respectively).

**Conclusions:**

In women with prior myalgia-related statin intolerance, vitamin D levels were significantly lower in women who remained on alternative day dosing compared to those who were tolerant of daily dosing.

## Background

Cardiovascular disease (CVD) is the single largest killer of women, [1] and more women than men die each year despite advancements in life-saving therapies [2]. In fact, patients with a total blood cholesterol level greater than 200 mg/dL have a two-fold risk of developing CVD. 3-Hydroxy-3-methylglutaryl-coenzyme A (HMG-CoA) reductase inhibitors (statins) are currently the most effective treatment for lowering total cholesterol (TC), calculated low density lipoprotein (LDL-C), and reducing atherosclerotic cardiovascular risk (ASCVD) [3]. With a heightened focus on reducing ASCVD risk, statin compliance is more important than ever in reducing cardiovascular events.

Compliance is often limited by myopathic pain symptoms. Approximately 1–2 % of patients will experience myalgias with statin therapy that can occur years after initiation of therapy [4]. In an earlier study, we demonstrated that women who experience myalgias with statin use can be put on an alternate day statin dosing regimen that is effective in reducing TC and LDL-C and decreases the incidence of myalgias [5].

The pathophysiologic mechanisms hypothesized to play a role in the development of statin-induced myalgias include competition at the cytochrome P-450 (CYP3A4) enzyme [6], deficiency of mitochondrial enzyme CoQ [7, 10] decreased plasma clearance in older patients [8], and/or vitamin D deficiency [6]. Myalgias are defined as an unexplained muscle discomfort often described as “flu-like symptoms in the setting of normal creatine kinase levels [9]. Patients may describe these symptoms as muscle aches, soreness, stiffness, muscle tenderness and cramps with exercise or immediately after exercise [9]. Myalgias are generally the first manifestation of vitamin D deficiency [6]. Due to the growing concern regarding the prevalence and significance of vitamin D deficiency [10, 11], we hypothesized that women who are unable to tolerate daily dosing statin therapy have lower vitamin D levels than those who are able to tolerate daily dosing.

## Methods

**Fig. 1:**
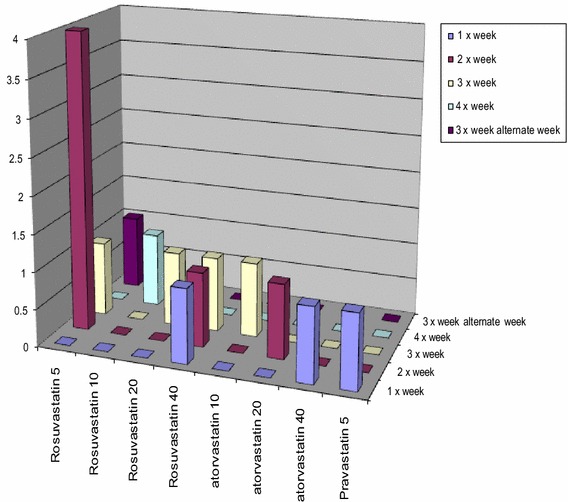
Prescribed dosing of statins used

We conducted a retrospective clinical chart review on 20 female patients from a tertiary chest pain center in 2008–2013. All women had an indication to be on statin therapy and had developed statin-induced myalgias. None of the women had a prior history of muscle related myalgias prior to statin therapy. According to clinical care practice, women with statin-induced myalgias were switched to a different statin and placed on an alternative day statin dosing regimen (simvastatin, atorvastatin, pravastatin, rosuvastatin, fluvastatin XL, or pitavastatin). Patients started low to moderate-intensity [3] statin therapy twice weekly, Mondays and Thursdays regimen for 4 weeks, and then titrated up 1 day per week as tolerated until either daily dosing was achieved or the patient experienced their prior myalgia pain. In that case, the patient was taken back to the previous tolerated alternative day dosing regimen (Fig. 1). Alternative day statin dosing was defined as any regimen less than daily. Patients included in the study were those with documented history of being placed on an alternative day statin dosing regimen with a corresponding vitamin D level drawn within 3 months of clinic visit. Laboratory data regarding vitamin D levels, creatinine kinase, hepatic transaminases, and fasting lipid panel were collected from chart review. Information regarding prior failed statin history, supplement use, cardiac risk factors, and demographic data was all obtained from chart review, which was approved by the Institutional Review Board at Cedars-Sinai Medical Center. Data were analyzed using a t test.

### Ethical aspects

This study conforms to the principles outlined in the Declaration of Helsinki and was approved by the Cedars-Sinai Institutional Review Board in Los Angeles, California, United States (Pro00023187). The study was registered at ClinicalTrials.gov (NCT01568255).

## Results

The study group mean age was 61 ± 13 years old and the mean body mass index (BMI) was 27 ± 7 kg/m^2^. The majority of women were Caucasian (17 women) and the remainder were African American (3 women). All women had a CVD indication to be on statin therapy. In regards to additional CVD risk factors, 11 women had hypertension, 10 women had a significant family history of premature coronary artery disease (CAD), and 6 women had known CAD, 10 women had a previous or active smoking history and none had diabetes. Women were further subdivided demographically as either alternative day statin dosing (n = 16) or daily dosing (n = 4) (Table 1). Only 5 women were taking non-prescription strength over-the-counter vitamin D supplements 1000–2000 IU at the time data was collected regarding vitamin D level and statin-induced myalgias.

**Table 1:** Demographic data

Baseline characteristics	Daily dosing (n = 4)	Alternate day dosing (n = 16)	*P* value*
Age (years ± SD)	61.8 ± 17.5	60.4 ± 11.8	0.9245
Hypertensive	50 %	56.3 %	1
CAD	25 %	35.7 %	1
Diabetes mellitus	0	0	–
History of smoking	100 %	40 %	0.0867

* Wilcoxon rank sum test was used for age. Fisher’s Exact test for other variables

Of the 20 women in the study, 16 (76 %) remained on alternative day statin dosing due to myalgias. On average, women had intolerance to at least 2 previous statins before finding a regimen that left them pain free. Wilcoxon rank sum tests between dosing groups showed no significant differences in total cholesterol on statin daily statin dosing vs. alternative day statin dosing (p = 0.5712), or LDL on daily statin dosing vs. alternative day statin dosing (p = 0.4367).

**Fig. 2:**
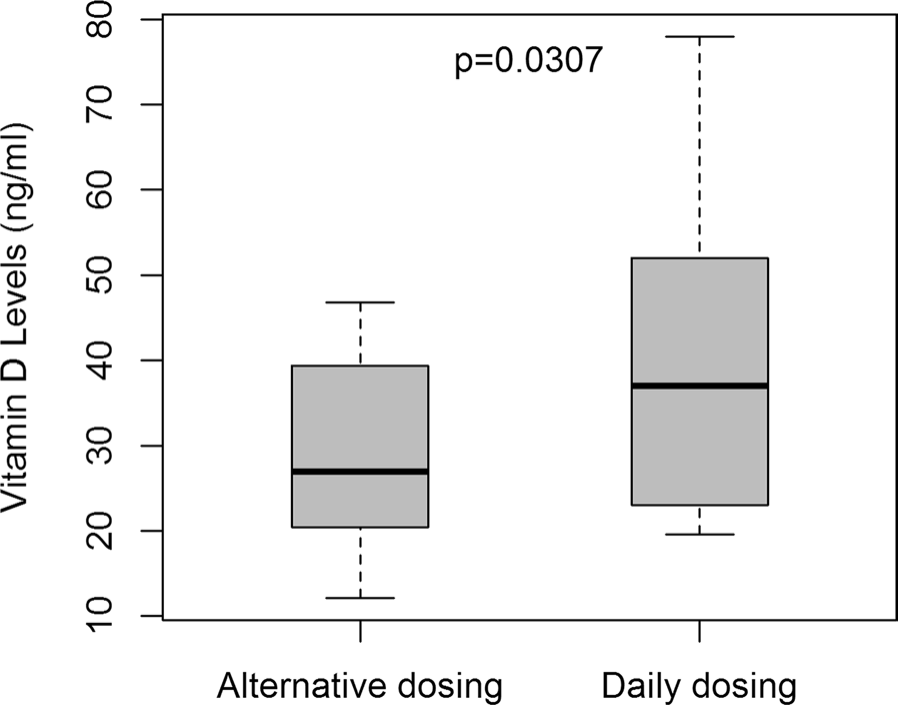
Vitamin D levels in alternative dosing vs. daily dosing in patients with statin-related myalgias

Overall, the mean 25-OHD level for the total group was 32.78 ± 15.62. Women who remained on alternative day statin dosing (n = 16) compared to women on daily dosing (n = 4) had a significantly lower group mean 25-OHD (mean 29.09 ± 11.23 vs. 47.5 ± 23.53 ng/ml t test p = 0.0307 respectively) (Fig. 2).

## Discussion

We report that women with statin myalgias and unable to tolerate daily statin dosing had significantly lower vitamin D levels compared to women who were able to achieve daily statin dosing. This pilot, cross-sectional, retrospective study supports previous anecdotal observations that vitamin D deficiency may be associated with statin-induced myalgias.

A systematic review and meta-analysis by Michalska-Kasiczak et al. supports our findings [11]. Their compilation of 2420 statin treated patients observed that vitamin D plasma concentrations were significantly lower in patients with statin-associated myalgias compared to those not experiencing myalgias [11]. Three published papers have previously suggested a positive association between vitamin D deficiency and statin-induced myalgias [6, 12, 13]. One clinical study conducted by Ahmed et al. [13] studied this association by treating vitamin D deficient patients with statin-induced myalgias with 50,000 IU of ergocalciferol. Overall, 92 % of patients had resolution of myalgia symptoms after repletion of vitamin D levels when challenged with the same statin used at study entry. However, the lack of a control arm prevents differentiation between a true therapeutic role of ergocalciferol versus a placebo effect.

The questionable relationship between vitamin D deficiency and statin-induced myalgias was brought under scrutiny in a Letter to the Editor published in the April 2011 issue of Atherosclerosis [14]. Backes et al. compared vitamin D levels of a group of 72 patients intolerant to a median of 3 different statin medications to a group of 57 patients with no previous history of statin intolerance and found no difference between groups [14]. While these findings are inconsistent with ours, this may be because our entire study population of 20 women had failed an average of 2 statins. We made comparisons of vitamin D levels within this group of intolerant patients, unlike Backes et al. who compared the group of intolerant statin users to patients who had never experienced myalgias.

There are several hypotheses to explain this relationship between vitamin D deficiency and statin-induced myalgias. As a patient becomes deficient in vitamin D they may develop osteomalaci myopathy. Low vitamin D stores in these patients are associated with increased body sway, decreased muscular strength, changes in gait, difficulties in rising from a chair, inability to ascend stairs and diffuse muscle pain [15, 16]. These symptoms of musculoskeletal pain appear to be similar in nature to the muscle pain, or myalgias, associated with statin use [13]. An alternate theory involves competition of the CYP3A4 isozyme that is both responsible for statin metabolism and vitamin D activation. It is theorized that in vitamin D deficient states, CYP3A4 preferentially hydroxylates 25-OHD instead of metabolizing statins, leading to statin-induced toxicity which may result in myalgias [4, 6, 7]. Statins that most commonly cause myalgias (simvastatin and atorvastatin) inhibit the CYP 3A4 system. Finally, age-related changes may be playing a role in the association of vitamin D deficiency and myalgias in the setting of statin use. It is well established that increasing age is a risk factor for developing statin-induced myalgias [3]. Early in vitro studies have shown that vitamin D receptors (VDR) on muscle cells have been shown to decrease with age [15, 16, 17]. Because our study population has a mean age of 60 ± 13 years of age, it may be that the women we observed have fewer VDR’s per muscle cell which may lead to decreased nuclear receptor activation causing poor muscle strength and/or function [15, 16–17].

### Limitations

Because our pilot study was a cross-sectional, retrospective study, conclusions regarding definitive linkage and causality cannot be drawn. Patients were not on a standardized statin regimen; therefore, it may be that a certain statin did not interact with vitamin D pathways resulting in the absence of myalgias. Furthermore, due to the small sample size, individual statins could not be assessed against vitamin D to identify independent associations; however, even with the small sample size, a statistically significant relationship was seen between two groups. There was a wide range in vitamin D levels and smaller numbers of women, the influence of the highest sample is larger and could have possibly affected the significance. We have taken this into consideration and have designed a larger, prospective, randomized, double blind study of 50,000 IU of ergocalciferol versus placebo.

## Conclusions

Our study findings support earlier suggestions of a positive association between vitamin D deficiency and statin-induced myalgias. More work needs to be done to define this relationship and uncover the mechanistic pathway for the relationship observed. To that end, we are conducting a randomized, double blinded placebo controlled interventional trial to test the hypothesis that vitamin D supplementation improves statin tolerance in subjects with vitamin D deficiency.

## Abbreviations

BMI: body mass index
CVD: cardiovascular disease
CAD: coronary artery disease
CYP3A4: cytochrome P-450
25-OHD: 25 hydroxyvitamin D
VDR: vitamin D receptor
